# Supplementary material for: Lafora Disease: A Case Report and Evolving Treatment Advancements
Source: Brain Sci. 2023 Dec 6;13(12):1679. doi: 10.3390/brainsci13121679 (PMC10742041; doi:10.3390/brainsci13121679)
Supplement: Supplementary file 1 [file brainsci-13-01679-s001.zip › Lafora Supplementary Data.pdf]

Lafora Disease: A Case Report, Myopathology, and Update on Advancements in Treatments

SUPPLEMENTARY DATA

Supplementary Materials and Methods
Supplementary Figure S1 Family Pedigree and Molecular Findings
Supplementary Video Video of the Patient displaying myoclonic seizures.

Supplementary Materials and Methods

Histological and immunohistochemical analysis of muscle biopsy

Tissue specimen was frozen in isopentane-cooled liquid nitrogen and processed according to standard techniques. For histological analysis, 8 µm-thick cryosections were picked and processed for routine staining with Hematoxylin and Eosin (H&E), Modified Gomori Trichrome (MGT), myosin ATPase (pH 9.4-4.6-4.3), cytochrome c oxidase (COX), succinate dehydrogenase (SDH), phosphatase acid, NADH, Oil Red O, Periodic Acid Schiff (PAS). Images fields were acquired using optical microscope Leica DM4000B equipped with DFC420C camera.

Electron microscopy of muscle biopsy

For ultrastructural examination a small part of muscle sample was fixed in 2.5% glutaraldehyde (pH 7.4), post fixed in 2% osmium tetroxide and then, after dehydration in a graded series of ethanol, embedded in Epon's resin. Finally, ultrathin sections were stained with lead citrate and uranyl acetate and examined with Zeiss EM109 transmission electron microscope.

Molecular Studies

After written informed consent, genomic DNA was extracted from peripheral blood samples of proband and parents using standard procedures. The exonic regions and flanking splice junctions of the genome were captured using the Clinical Constitutional Panel 17 (Agilent Technologies, Santa Clara, CA). Sequencing was done on a NextSeq500 Illumina system with 150bp paired end reads. Reads were aligned to human genome build GRCh37/UCSC hg19 and analyzed with the IGV software. Variants prioritization was performed by using the Alissa Platform (Agilent).

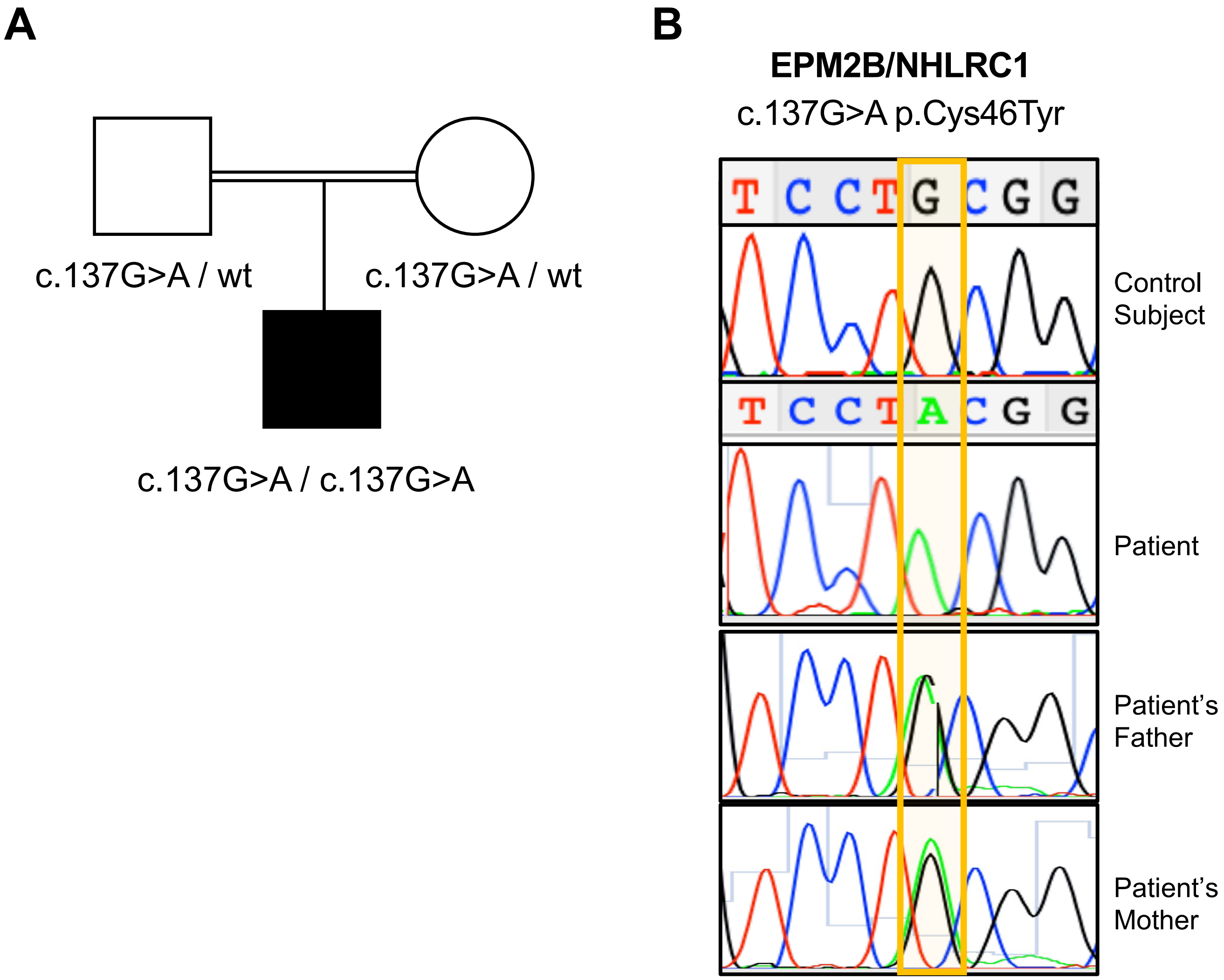

Supplementary Figure S1. (**A**) Pedigree of the family. The patient is indicated by black symbol. Genotype of each investigated subjects is indicated under the corresponding symbol. (**B**) Electropherograms showing the genotype of the position c.137 of *EPM2B*/*NHLRC1* gene in the pedigree.
